# Period of Isolation of School Children for Contagious Diseases

**Published:** 1899-08

**Authors:** 


### PERIOD OF ISOLATION OF SCHOOL CHILDREN FOR CONTAGIOUS DISEASES.

According to the *Revue Mensuelle des Maladies de l'Enfance*, August, 1898, the Medical Council of the Russian Empire has established the following periods of time for the isolation of school children who have been exposed to an infectious disease or have themselves suffered from such a disease:

SCARLATINA.—After exposure, and without development of symptoms, an isolation of from twelve to fourteen days is required. A child that has been ill may be allowed to return to school six weeks after the appearance of the eruption, provided there is after a time no trace of desquamation.

MEASLES.—Fifteen days after exposure; or, in case the disease has been present, four weeks from the beginning of the eruption, if there is no trace of desquamation.

RUBELLA.—Sixteen days; or after two weeks from the beginning of the eruption.

VARICELLA.—Seventeen days; or after the fall of the crusts.

PERTUSSIS.—Fifteen days to twenty days; or after six weeks from the beginning of the cough, if kinks have ceased, and there is no expectoration.

MUMPS.—Twenty-two days; or after three weeks from the beginning of the parotid swelling.

DIPHTHERIA.—Seventeen days; or three weeks after recovery, and after the disappearance of hyperemia of the pharynx, larnyx, and nose; if bacteriological examination is possible, only after the disappearance of the bacilli.

VARIOLA.—Fourteen days; or after the fall of the crusts.

All convalescent patients should receive two or three warm baths at 35 degrees C.—*American Journal of the Medical Sciences, December, 1898.*

